# In vitro and in vivo activity of hyperimmune globulin preparations against multiresistant nosocomial pathogens

**DOI:** 10.1007/s15010-014-0706-1

**Published:** 2014-11-27

**Authors:** F. S. Rossmann, A. Kropec, D. Laverde, F. R. Saaverda, D. Wobser, J. Huebner

**Affiliations:** 1 Division of Infectious Diseases, Department of Medicine, University Hospital Freiburg, Freiburg im Breisgau, Germany; 2 Faculty of Biology, Albert-Ludwigs-University Freiburg, Freiburg University, Freiburg im Breisgau, Germany; 3 Division of Pediatric Infectious Diseases, Department of Pediatrics, Dr. von Hauner Children’s Hospital, Ludwig-Maximilians University, Munich, Germany; 4 German Center for Infection Research (DZIF), Partnersite Munich, Munich, Germany

**Keywords:** Immune globuline preparations, IgG, IgM, Nosocomial pathogens, Opsonic killing, Protective efficacy, Animal model

## Abstract

**Purpose:**

We compared different immunoglobulin preparations containing IgG (Intraglobin/Intratect) or a mixture of IgG, IgA, and IgM (Pentaglobin) to assess the opsonic and protective efficacy of human immunoglobulin preparations against multiresistent nosocomial pathogens.

**Materials and methods:**

Clinical isolates of *E. coli*, *Klebsiella pneumoniae*, *Pseudomonas aeruginosa*, *Enterococcus faecalis*, *Enterococcus faecium*, and *Staphylococcus aureus* were tested by opsonophagocytic assay using immunologobulin preparations at dilutions usually obtained in patients. The target antigens of opsonic antibodies were characterized by opsonophagocytic inhibition assays, and the protective efficacy in vivo was tested in a mouse bacteremia model as previously described.

**Results:**

All strains were killed to at least 50 % by Pentaglobin. One *P. aeruginosa* strain was not efficiently killed by Intraglobin (23 %) but the other strains were killed by Intraglobin to a similar degree compared to Pentaglobin. Opsonic IgG antibodies against *E. faecalis* were directed against LTA, while opsonic antibodies in Pentaglobin were primarily directed against other cell wall carbohydrates. In a mouse bacteremia model, Pentaglobin was more protective than Intratect against *Staphylococcus aureus*, while Intratect reduced colony counts better than normal rabbit serum or saline.

**Conclusions:**

All tested human immunoglobulin preparations contain opsonic and protective antibodies against targets present on multiresistant Gram-positive and Gram-negative bacteria. Enrichment of these preparations with IgM increases the protective efficacy against some strains, probably due to antibodies directed against cell wall carbohydrates.

## Introduction

Multiresistant nosocomial pathogens often cause life-threatening infections that are sometimes untreatable with currently available antibiotics and are therefore one of the most serious problems in modern medicine. A recent report from the Centers for Disease Control and Prevention (CDC) estimates that in the US about two million people acquire infections with resistant bacteria, and that probably about 23,000 patients die each year as a direct consequence of these infections [1]. Gram-positive bacteria account for a large proportion, and staphylococci and enterococci are the most important bacterial species causing these mostly hospital-acquired infections that often lead to extended hospital stay and excess mortality. Multiresistant *Staphylococcus aureus* cause pneumonia, skin, wound, bloodstream and surgical site infections. About 80,000 *S. aureus* infections have been reported in the US per year with about 12,000 deaths caused by bacteria resistant to methicillin (MRSA) [1]. High rates are also seen for enterococci, mainly *Enterococcus faecium* resistant to vancomycin (VRE) causing bloodstream infections, urinary tract infections, and foreign-body infections (e.g., catheters, stents, CNS shunts, artificial heart valves, etc.) mostly in immunocompromised patients [2–4]. For the US, it is estimated that about 66,000 enterococcal infections occur each year, and about 20,000 of these are due to multiple-drug resistant (i.e., VRE) with about 1,300 deaths per year [1]. Multiresistance in Gram-negative bacteria is mostly due to extended-spectrum betalactamases (ESBL) or carbapenemases. ESBL-producing *Enterobacteriaceae* are increasingly isolated in patients and even otherwise healthy individuals [5]. These bacteria can cause bloodstream infections and are responsible for approximately 26,000 cases and 1,700 deaths per year [1]. Especially worrisome is a novel threat associated with resistance determinants against carbapenems, currently the antibiotics with the broadest spectrum. In the US, more than 9,000 healthcare-associated infections are caused by carbapenem-resistant *Enterobacteriaceae* (*K. pneumoniae and E. coli*) and 600 deaths result from these infections each year [1]. Similar trends and incidences have been reported in Europe (http://www.ecdc.europa.eu/en/activities/surveillance/EARS-Net/Pages/index.aspx).

Immunotherapy, either through active immunization, or by passive immunotherapy, is among the most promising alternative approaches to fight these strains [4, 6]. While the development of novel vaccines against multiresistant nosocomial pathogens is pursued by many of the leading pharmaceutical companies, no preparation has yet been introduced into the market [7]. Passive immunotherapy, either through monoclonal antibodies, or through hyperimmunoglobulins, may be an attractive addition to the currently available treatment options. However, no good data exist to support the usage of these preparations to prevent and/or treat infections in hospitalized patients.

Intravenous application of immunoglobulins from healthy volunteers has been used for a number of indications, e.g., immunoglobulin substitution (i.v.) in patients with agammaglobulinemia, and as a supportive therapy for bacterial infections, Kawasaki disease, or Guillain-Barré syndrome. Most currently available antibody preparations contain only IgG and several studies could not demonstrate a benefit of these antibodies in the prevention and treatment of bacterial infections. However, in the initial immune response against bacterial pathogens, IgM plays an important role and the production of a compound containing not only IgG but also IgA and IgM may therefore offer the advantage of an “innate” immune protection against bacteria [8]. Here, we investigate and compare the efficacy of protection of different antibody preparations with and without IgM and demonstrate that IgM provides a better protection in vivo and in vitro against some nosocomial pathogens.

## Materials and methods

### Bacterial strains, antibody preparations, and antigens

Bacterial strains, antibodies, and antigens used in this study are listed in Table 1. Immunoglobulin preparations were obtained from Biotest, and antigens were either produced in our lab according to previously described methods (i.e., LTA [9] or purchased from Sigma (St. Louis, MO).

**Table 1:** Bacterial strains, antigens and antisera used in this study

Bacteria	Origin	Reference/provider
*E. faecalis* 12030	Clinical isolate, Cleveland, OH, (gift from D. Shlaes)	[14]
*E. coli* 4263	Outbreak isolate, strain collection University Hospital Freiburg	
*E. faecium* 1162	Isolated from blood in the Netherlands, CC17	[27]
*K. pneumonia* 1436	Outbreak isolate, strain collection University Hospital Freiburg	
*K. pneumonia* 1437 ESBL	Outbreak isolate, strain collection University Hospital Freiburg	
*P. aeruginosa* 2790 carbapenemase resistant	Outbreak isolate, strain collection University Hospital Freiburg	
*S. aureus* LAC	CA-MRSA (USA300)	[28]
VRSA-I	Vancomycin-resistant *S. aureus* (NARSA) http://www.niaid.nih.gov/labsandresources/resources/dmid/narsa/Pages	[29]
Antigens		
LTA	Lipoteichoic acid from *E. faecalis* 12030	[9]
LTA	Lipoteichoic acid from *Streptococcus* (*Enterococcus*) *faecalis*	Sigma
Antisera		
Intratect	Contains only IgG (100 mg/mL)	Biotest
Intraglobin	Contains only IgG (50 mg/mL), same donor pool as Pentaglobin (discontinued)	Biotest
Pentaglobin	6 mg IgM, 6 mg IgA and 38 mg IgG (50 mg/mL), same donor pool as Intraglobin	Biotest

### Opsonophagocytic assay

Opsonophagocytic killing was assessed as described by Theilacker et al. [10] using 1.7 % baby rabbit serum (Cedarlane) as complement source, and rabbit serum raised against purified lipoteichoic acid (anti-LTA) from *E. faecalis* 12030 as positive control [11–13]. Bacteria were incubated and grown to mid-exponential phase (OD_650nm_ = 0.400). Equal volumes of bacterial suspension (2.5 × 10^7^ per mL), leukocytes (2.5 × 10^7^ per mL), complement source (1.7 % final concentration), and either anti-LTA rabbit serum, immunoglobulin preparations or heat-inactivated immune rabbit serum (as control) were combined and incubated on a rotor rack at 37 °C for 90 min. After incubation, colony-forming units (CFUs) surviving in the tubes with bacteria were quantified by agar culture of serial dilutions. Percentage of killing was calculated by comparing the colony counts at 90 min (*t*90) of a control without PMNs (PMN^neg^) to the colony counts of a tube that contained all four components using the following formula: {[(mean CFU PMN^neg^ at *t*90) − (mean CFU at *t*90)]/(mean CFU PMN^neg^ at *t*90)} × 100.

### Proteinase K and sodium meta-periodate treatment for inhibition experiments

Bacterial strain *E.* *faecalis* 12030 was cultured overnight in TSB, harvested by centrifugation (8,000 rpm, 10 min, 4 °C), and washed three times with PBS. Treatment of bacterial cells with proteinase K was performed as described previously [14]. In brief, bacterial cells (≈10^9^ cfu/mL) were incubated with proteinase K (Sigma) at a final concentration of 0.1 mg/mL and 5 mM calcium chloride at 54 °C during 4 h. Treated cells were heat inactivated at 65 °C for 1 h, washed three times with PBS, and adjusted to a final concentration of 2.5 × 10^11^ cfu/mL in PBS for the opsonophagocytic inhibition assay. For sodium meta-periodate treatment [15], bacterial cells (≈10^9^ cfu/mL) were incubated with sodium meta-periodate at a final concentration of 1 M for 24 h at room temperature in the dark. Sodium meta-periodate was neutralized with an excess of ethylene glycol at a final concentration of 2 M. Treated cells were washed three times with PBS and adjusted to a final concentration of 2.5 × 10^11^ cfu/mL in PBS for opsonophagocytic inhibition assay.

### Opsonophagocytic inhibition assay

For inhibition studies, either pre-treated bacterial cells or lipoteichoic acid was used as inhibitor. Pentaglobin (50 mg/mL) and Intratect (100 mg/mL) were diluted 1:25 and incubated for 60 min at 4 °C with an equal volume of a solution containing 1.25 × 10^11^ cfu/mL of treated bacterial cells or 100, 20, 4 or 0.08 µg/mL of either lipoteichoic acid purified in our lab from *E. faecalis* 12030 or lipoteichoic acid from *S. faecalis* (*E. faecalis*) purchased from Sigma (St. Louis, Mo.). Subsequently, the respective mixtures of antibody and inhibitor was used in the opsonophagocytic assay (OPA) as described above. Inhibition assays were performed at serum dilutions yielding 50–80  % killing of the inoculum without the addition of the inhibitor. The percentage of inhibition of opsonophagocytic killing was compared to controls without inhibitor.

### Animal experiments

The protective efficacy of the monoclonal antibodies was tested against CA-MRSA strain LAC in a mouse bacteremia model as described previously [11]. Five female BALB/c 6- to 8-week-old mice per group (Charles River Laboratories Germany GmbH) were infected by i.v. injection of CA-MRSA (5.0 × 10^7^ cfu) via the tail vein. Antibodies were given 48 and 24 h i.p. prior to bacterial challenge. Mice were killed 48 h after infection and organs (livers and kidneys) were aseptically removed, weighted and homogenized. Bacterial counts were enumerated after overnight incubation by plating serial dilutions on tryptic soy agar (TSA) plates. Statistical significance was assessed by Mann–Whitney test [12].

All animal experiments were performed in compliance with the German animal protection law (TierSchG). The animals were housed and handled in accordance with good animal practice as defined by FELASA and the national animal welfare body GV-SOLAS. The animal welfare committees of the University of Freiburg (Regierungspraesidium Freiburg Az 35/9185.81/G-12/070) approved all animal experiments. The institutional review board of the University of Freiburg approved the study protocol.

## Results

To assess the role of the IgM component with regard to opsonic killing, we compared Pentaglobin (IgG, IgM and IgA) with Intraglobin (only IgG). For the production of Intraglobin the same donor pool was used as for Pentaglobin.

**Fig. 1:**
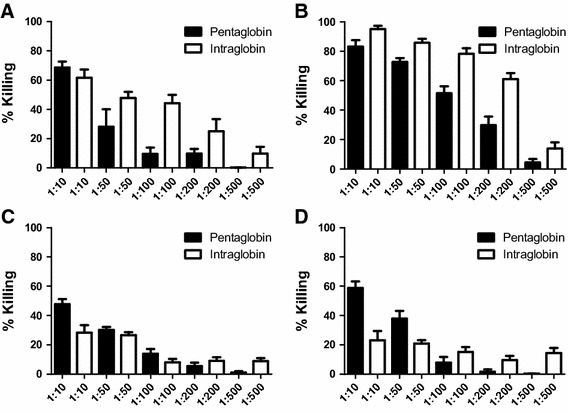
Opsonophagocytic killing of ESBL-producing strain *K. pneumoniae* 1437 (**a**), *K. pneumoniae* 1436 (**b**), *E. coli* 4263 (**c**), and carbapenem-resistant *P. aeruginosa* 2790 (**d**). Opsonophagocytic assay was performed as described by Theilacker et al. using baby rabbit serum as complement source. IVIG preparations show significant opsonic killing against both *K.* *pneumoniae* strains. Higher killing against *E. coli* and *P. aeruginosa* was observed using Pentaglobin in comparison to Intraglobin. Killing is measured at dilutions of 1:10, 1:50, 1:100, 1:200 and 1:500. Killing was dose dependent with both immunoglobulin preparations

Comparing the IgM-containing compound with the IgG compound against Gram-negative bacteria, significant killing was seen against ESBL-producing strain *K. pneumoniae* 1437 at a dilution of 1:10 (i.e., 68 % killing with Pentaglobin and 61 % with Intraglobin) (Fig. 1a). Higher killing could be observed in *K. pneumoniae* 1436, which at a dilution of 1:10 showed the best killing using Pentaglobin (83 %) and Intraglobin (95 %) (Fig. 1b). Clearly, less killing was observed in *E. coli* 4263 (47 %) and *P. aeruginosa* 2790 (58,8 %) using Pentaglobin, and even less killing was seen with Intraglobin (28 % for *E. coli* 4263 and 23 % for *P. aeruginosa* 2790) (Fig. 1c, d). Overall, the IVIG preparation containing IgM shows higher killing rates in comparison to pure IVIG preparation Intraglobin against Gram-negative bacteria (Fig. 1).

**Fig. 2:**
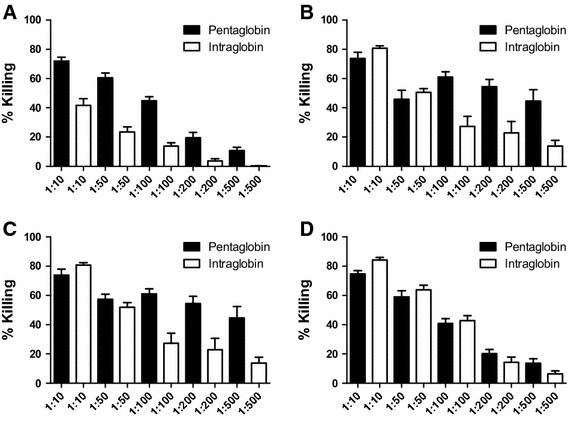
Opsonophagocytic killing of VRSA-I strain (**a**), CA-MRSA LAC (**b**), *E. faecalis* 12030 (**c**), and *E. faecium* 1162 (**d**). Opsonophagocytic assay was performed as described by Theilacker et al. using baby rabbit serum as complement source. IVIG preparations show significant opsonic killing against all tested strains. Killing is measured at dilutions of 1:10, 1:50, 1:100, 1:200 and 1:500. Killing was dose dependent with both immunoglobulin preparations

To compare the IgM-containing compound with the pure IgG preparation against Gram-positive bacteria, killing rates of Pentaglobin and Intraglobin were also compared against several nosocomial Gram-positive pathogens. Killing against vancomycin-resistant *S. aureus* was significantly higher with Pentaglobin (71 %) at a 1:10 dilution in comparison to Intraglobin (41 %) (Fig. 2a). However, opsonic killing against CA-MRSA, *E. faecalis* as well as *E. faecium* was higher with Intraglobin (80–84 %) in comparison to Pentaglobin (73–74 %) (Fig. 2).

**Fig. 3:**
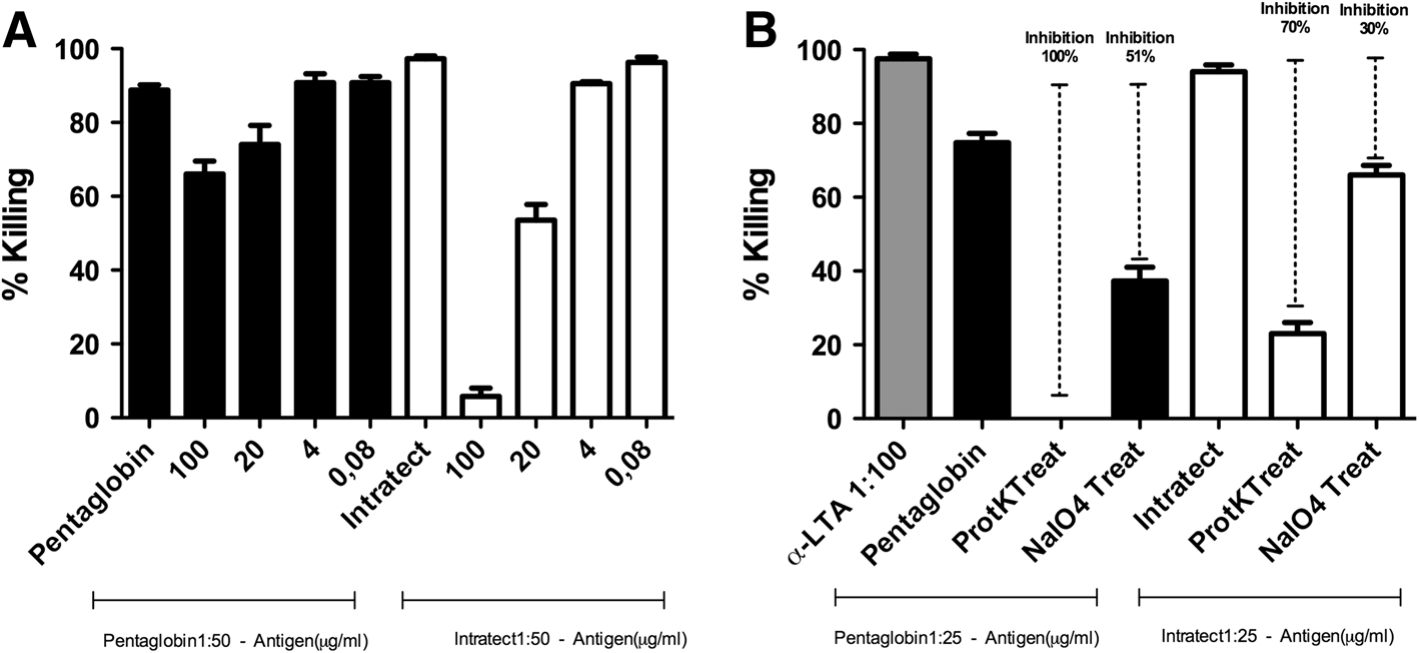
Opsonophagocytic inhibition assay comparing the target of Pentaglobin and Intratect. **a** Lipoteichoic acid is the target of opsonic antibodies against *E. faecalis* 12030 in Intratect, while Pentaglobin is only partially directed against LTA. **b** Opsonophagocytic inhibition assay after treating *E. faecalis* 12030 with proteinase K or NaIO_4_. Opsonic activity of Intratect is significantly inhibited when bacteria are treated with proteinase K (70 %) and inhibited by 30 % when bacteria are treated with NaIO_4_. Pentaglobin is only partially inhibited with the cells treated with NaIO_4_ indicating that the majority of the antibodies are directed against polysaccharides and some are directed against protein antigens. The *gray bar* shows Killing against *E. faecalis* 12030 when sera raised against LTA from *E. faecalis* 12030 were used and represent the positive control within this experiment. Intratect and Pentaglobin were diluted 1:25 for the inhibition assay

To assess the bacterial targets of the opsonic antibodies in the various immunoglobulin preparations, Pentaglobin and Intratect (the current commercially available IgG preparation), were used in an opsonophagocytic inhibition assay [16]. Since our previous results indicated that the glycerol phosphate backbone of LTA is the predominant target of protective antibodies against Gram-positive bacteria [12], we used an LTA preparation for absorptions, indicating that opsonic IgG antibodies against *E. faecalis* in Intratect are directed against this epitope. Using the IgM-containing compound, only minimal absorption of killing was observed with purified LTA, indicating that the majority of opsonic antibodies in this preparation are not directed against this antigen (Fig. 3a). Opsonic killing activity of Intratect was inhibited with bacterial cells either treated with sodium meta-periodate or proteinase K, suggesting that about 70 % of the antibodies of Intratect are directed against a polysaccharide target and 30 % against bacterial protein antigens. In contrast, opsonic antibodies in Pentaglobin are only partially absorbed by bacterial cells treated with NaIO_4_ but quite efficiently by bacteria treated with proteinase K. This indicates that the majority of opsonic antibodies in this preparation are directed against carbohydrate antigens, while a smaller number of opsonic antibodies are also directed against protein antigens (Fig. 3b).

**Fig. 4:**
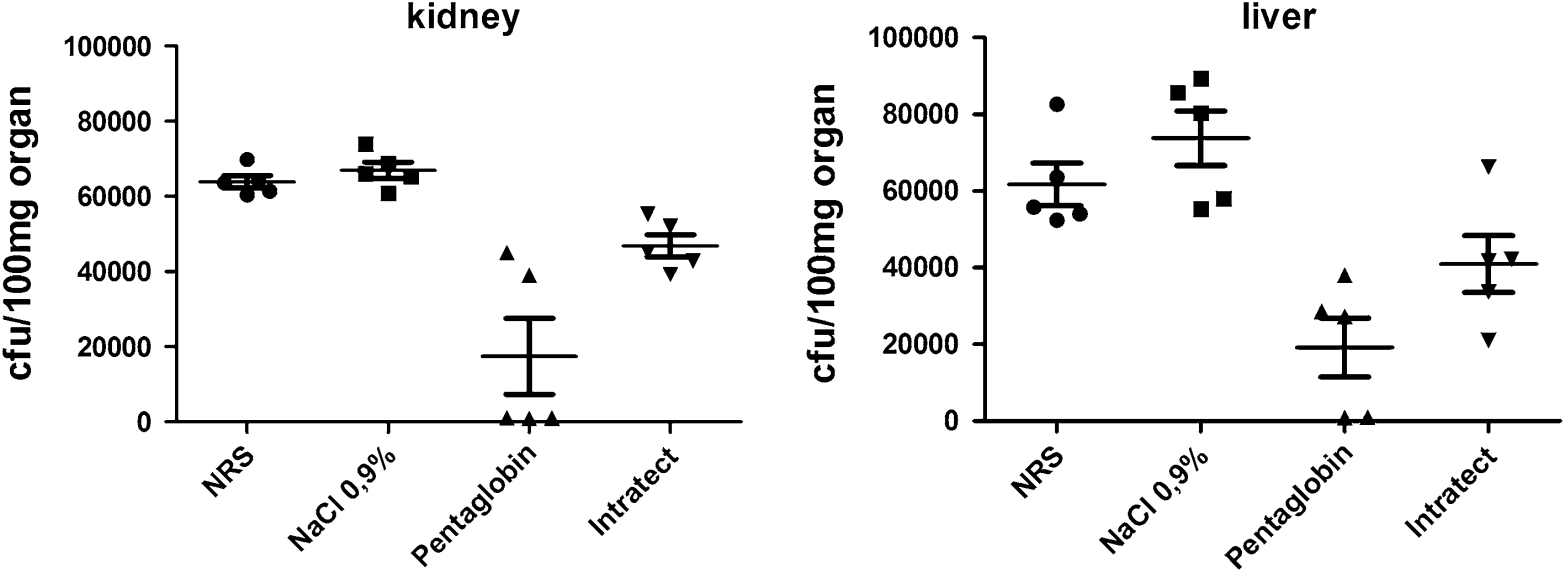
Mouse bacteria sepsis model using CA-MRSA. Bacteria mouse sepsis was performed as described by Bao et al. The numbers of bacteria recovered from the kidney (**a**) and liver (**b**) of mice infected i.v. with *S. aureus* LAC were significantly lower (1.0 × 10^4^ cfu/g kidney and 1.9 × 10^4^ cfu/g liver) when animals received the IgG/IgM preparation Pentaglobin (300 mg/kg) compared to animals treated with Intratect (4.5 × 10^4^ cfu/g kidney and 4.0 × 10^4^ cfu/g liver)

Using a previously described mouse sepsis model, we assessed the protective efficacy of the two antibody preparations in vivo (Fig. 4). Numbers of bacteria in liver and kidney of animals infected with MRSA strain LAC were significantly lower (1.0 × 10^4^ cfu/g kidney and 1.9 × 10^4^ cfu/g liver) when animals received the IgG/IgM preparation Pentaglobin (300 mg/kg) compared to animals treated with Intratect (4.5 × 10^4^ cfu/g kidney and 4.0 × 10^4^ cfu/g liver).

## Discussion

The concept of using polyclonal human antibody preparations to treat and/or prevent bacterial infections has been pursued for a long time [17, 18]. However, results so far are rather disappointing. A large meta-analysis about the usage of IVIG in preterm neonates to prevent infectious complications found a rather low reduction of about 3 % in sepsis. However, this was not associated with a reduction in other clinically important outcomes, including mortality [19]. Similar observation has been made in other clinical settings [20].

The role of IgM is predominantly in the primary immune response of bacterial infections, and IgM eventually hypermutates and undergoes isotype switching [21]. Nevertheless, in many bacterial infections, the protective immune response is directed against carbohydrate antigens (such as capsular polysaccharides or glycoconjugates, such as teichoic acids) that are classic “T cell independent antigens” and do not lead to isotype switching and memory B cell generation [22]. These antibodies seem to be critically important for the first-line defense against common bacterial pathogens [8, 21].

Only one commercially available antibody preparation, Pentaglobin, contains significant amounts (12 %) of IgM. In one study, this compound significantly reduced mortality, but had no overall effect on ICU length of stay [23]. On the other hand, Tugrul and colleagues [24] did not see such an effect and concluded that Pentaglobin did not have a positive effect on morbidity, incidence of septic shock, and mortality. Berlot et al. [20] pointed out that in general, the timing of the application of IVIG preparations is probably related to efficiency, similar to broad-spectrum antibiotics. The effect of IgM may also not be only directed against bacterial pathogens but may in addition lead to a modified anti-inflammatory response, such as up-regulation of IL-10 or down-regulation of IL-1β [25]. The selection of the study population and the overall study design seem to be of importance, because there are strong indications that certain subgroups, such as patients with streptococcal toxic shock syndrome caused by group A streptococcus, will benefit [26], while other more heterogeneous study populations may not exhibit positive results.

In our study, both antibody preparations showed broadly cross-reactive antibodies against the most notorious multiresistant pathogens, leading to significant opsonic killing of 50 % and higher at concentrations of 1.25 mg/mL against most of the strains tested. This amount corresponds to the human situation where similar amounts of immunoglobulin should be obtained with the recommended doses of Pentaglobin or Intratect. Pentaglobin clearly showed better protection at lower dilutions (as low as 125 µg/mL), while Intraglobin did not show significant killing at these dilutions. Since the only difference between these preparations is the IgM (and IgA) content of Pentaglobin, this may indicate that the opsonic and protective antibodies are IgM.

At least against *E. faecalis*, the target of opsonic IgG antibodies seems to be lipoteichoic acid, since absorption with purified LTA abrogated killing of this strain. For Pentaglobin, absorption with LTA did not result in significant inhibition of killing, although the majority of killing was abrogated after absorption with proteinase-treated bacterial cells. This indicates that the targets of protective antibodies of both preparations differ to some extent, but are mainly directed against carbohydrate antigens (such as capsular polysaccharides). Killing in the opsonophagocytic assay is usually expressed in percent, although the effect is logarithmic. This explains the observation that inhibition with proteinase-K-treated bacteria treated with Na-periodate does not add up to 100 %. The animal model clearly shows protection against bacterial infections by both antibody preparations, although lower bacterial counts were observed with Pentaglobin, compared to Intratect. Together, our data indicate that human immunoglobulin preparations contain opsonic and protective antibodies against targets present on multiresistant Gram-positive and Gram-negative bacteria. Enrichment of these preparations with IgM increases the protective efficacy, probably due to antibodies directed against cell wall carbohydrates. Additional studies in well-defined clinical settings should confirm these findings.

## References

[1] Centers for Disease Control and Prevention CDC. Antibiotic resistance threats in the United States 2013 Internet www.cdc.gov 2013 cited 2014 Jul 3. pp. 1–114. Available from: http://www.cdc.gov/drugresistance/threat-report-2013/.

[2] Huebner J, Arciola CR (2007). Cave enterococcum!. Int J Artif Organs.

[3] Fabretti F, Huebner J (2005). Implant infections due to enterococci: role of capsular polysaccharides and biofilm. Int J Artif Organs.

[4] Koch S, Hufnagel M, Theilacker C, Huebner J (2004). Enterococcal infections: host response, therapeutic, and prophylactic possibilities. Vaccine.

[5] Talbot GH, Bradley JS, Edwards JE, Rice LB, Spellberg B (2009). Bad bugs, no drugs: no ESKAPE! an update from the Infectious Diseases Society of America. Clin Infect Dis.

[6] Fox JL (2013). Anti-infective monoclonals step in where antimicrobials fail. Nat Biotechnol.

[7] Shinefield H, Black S, Fattom A, Horwith G, Rasgon S, Ordonez J (2002). Use of a *Staphylococcus aureus* conjugate vaccine in patients receiving hemodialysis. N Engl J Med.

[8] Weller S, Braun MC, Tan BK, Rosenwald A, Cordier C, Conley ME (2004). Human blood IgM “memory” B cells are circulating splenic marginal zone B cells harboring a prediversified immunoglobulin repertoire. Blood.

[9] Theilacker C, Kaczynski Z, Kropec A, Fabretti F, Sange T, Holst O (2006). Opsonic antibodies to *Enterococcus faecalis* strain 12030 are directed against lipoteichoic acid. Infect Immun.

[10] Theilacker C, Sanchez-Carballo P, Toma I, Fabretti F, Sava I, Kropec A (2009). Glycolipids are involved in biofilm accumulation and prolonged bacteraemia in *Enterococcus faecalis*. Mol Microbiol.

[11] Hufnagel M, Koch S, Creti R, Baldassarri L, Huebner J (2004). A putative sugar-binding transcriptional regulator in a novel gene locus in *Enterococcus faecalis* contributes to production of biofilm and prolonged bacteremia in mice. J Infect Dis.

[12] Theilacker C, Kropec A, Hammer F, Sava I, Wobser D, Sakinc T (2012). Protection against *Staphylococcus aureus* by antibody to the polyglycerolphosphate backbone of heterologous lipoteichoic acid. J Infect Dis.

[13] Theilacker C, Kaczynski Z, Kropec A, Sava I, Ye L, Bychowska A (2011). Serodiversity of opsonic antibodies against *Enterococcus faecalis*—glycans of the cell wall revisited. PLoS One.

[14] Huebner J, Wang Y, Krueger WA, Madoff LC, Martirosian G, Boisot S (1999). Isolation and chemical characterization of a capsular polysaccharide antigen shared by clinical isolates of *Enterococcus faecalis* and vancomycin-resistant *Enterococcus faecium*. Infect Immun.

[15] Sava IG, Zhang F, Toma I, Theilacker C, Li B, Baumert TF (2009). Novel interactions of glycosaminoglycans and bacterial glycolipids mediate binding of enterococci to human cells. J Biol Chem.

[16] Kropec A, Sava IG, Vonend C, Sakinc T, Grohmann E, Huebner J (2011). Identification of SagA as a novel vaccine target for the prevention of *Enterococcus faecium* infections. Microbiology.

[17] Wilson PC, Andrews SF. Tools to therapeutically harness the human antibody response. Nat Rev Immunol. Nature Publishing Group; 2012;12:709–19. 10.1038/nri3285 PMC7097371 23007571

[18] Hufnagel M, Sixel K, Hammer F, Kropec A, Sava IG, Theilacker C, et al. Detection of opsonic antibodies against *Enterococcus faecalis* cell wall carbohydrates in immune globulin preparations. Infection. Springer Berlin Heidelberg; 2014;42:749–55. 10.1007/s15010-014-0628-y 24854332

[19] Ohlsson A, Lacy JB. Intravenous immunoglobulin for preventing infection in preterm and/or low birth weight infants. In: Ohlsson A, editor. Cochrane database of systematic reviews (Online). Chichester: John Wiley & Sons, Ltd; 2013;7:CD000361. 10.1002/14651858.CD000361.pub3 23821390

[20] Berlot G, Vassallo MC, Busetto N, Bianchi M, Zornada F, Rosato I (2012). Relationship between the timing of administration of IgM and IgA enriched immunoglobulins in patients with severe sepsis and septic shock and the outcome: a retrospective analysis. J Crit Care.

[21] Grönwall C, Vas J, Silverman GJ (2012). Protective roles of natural IgM antibodies. Front Immunol.

[22] Mond JJ, Lees A, Snapper CM (1995). T cell-independent antigens type 2. Annu Rev Immunol.

[23] Neilson AR, Burchardi H, Schneider H (2005). Cost-effectiveness of immunoglobulin M-enriched immunoglobulin (Pentaglobin) in the treatment of severe sepsis and septic shock. J Crit Care.

[24] Tugrul S, Ozcan PE, Akinci O, Seyhun Y, Cagatay A, Cakar N (2002). The effects of IgM-enriched immunoglobulin preparations in patients with severe sepsis [ISRCTN28863830]. Crit Care.

[25] Barratt-Due A, Sokolov A, Gustavsen A, Hellerud BC, Egge K, Pischke SE, et al. Polyvalent immunoglobulin significantly attenuated the formation of IL-1β in *Escherichia coli*-induced sepsis in pigs. Immunobiology. Elsevier GmbH; 2013;218:683–9. 10.1016/j.imbio.2012.08.268 22947599

[26] Norrby-Teglund A, Kotb M. Cytokine patterns in severe invasive group A streptococcal infections. In: Kotb M, Calandra T, editors. Cytokines and chemokines in infectious diseases handbook. Humana Press Inc; 2003. pp. 77–92.

[27] Heikens E, Bonten MJM, Willems RJL (2007). Enterococcal surface protein Esp is important for biofilm formation of *Enterococcus faecium* E1162. J Bacteriol.

[28] Pang YY, Schwartz J, Thoendel M, Ackermann LW, Horswill AR, Nauseef WM (2010). agr-Dependent interactions of *Staphylococcus aureus* USA300 with human polymorphonuclear neutrophils. J Innate Immun..

[29] Saravolatz LD, Pawlak J, Johnson LB (2012). In vitro susceptibilities and molecular analysis of vancomycin-intermediate and vancomycin-resistant *Staphylococcus aureus* isolates. Clin Infect Dis.

